# Tislelizumab plus lenvatinib for hepatocellular carcinoma with hypothyroidism. A case report and literature review

**DOI:** 10.3389/fimmu.2025.1630058

**Published:** 2025-10-31

**Authors:** Biaobiao Mao, Renya Jiang

**Affiliations:** ^1^ Jinhua Graduate Joint Training Base, Zhejiang Chinese Medical University, Hangzhou, China; ^2^ Department of Hepatobiliary Surgery, The Quzhou Affiliated Hospital of Wenzhou Medical University, Quzhou People’s Hospital, Quzhou, China

**Keywords:** hepatocellular carcinoma, lenvatinib, tislelizumab, hypothyroidism, literature review

## Abstract

This report presents the case of a 70-year-old female patient with hepatocellular carcinoma (HCC) who was hospitalized for fever of unknown origin. We confirmed the diagnosis of HCC with lymphadenopathy through needle biopsy. The patient experienced hypertension and malaise after therapy with tislelizumab plus lenvatinib. Additionally, there was a persistent decrease in T3 and T4 levels accompanied by elevated TSH levels. During treatment, tumor regression and notable shrinkage of enlarged lymph nodes met the criteria for surgical intervention. However, since the patient’s family declined surgery, the tumor subsequently progressed, resulting in clinical deterioration. This case highlights the uncommon occurrence of hypothyroidism after targeted immunotherapy in HCC patients and emphasizes the critical role of monitoring thyroid function in prognosis assessment. Conclusion: Hypothyroidism following combination therapy with tislelizumab and lenvatinib in HCC may indicate a favorable therapeutic response. Early surgical intervention is recommended once tumor shrinkage and symptom improvement occur.

## 1 Introduction

Hepatocellular carcinoma (HCC) is a significant global health challenge. It ranks as the third leading cause of cancer-related deaths worldwide and the second in China, raising substantial clinical concerns (1). Surgical resection is associated with a 5-year survival rate of more than 70%, making it the most effective treatment for early-stage HCC (2). Unfortunately, owing to the insidious onset of HCC symptoms, most patients are diagnosed at an advanced stage and/or are not suitable for surgical intervention, leading to a generally poor prognosis with a 5-year survival rate of only 10-20% (3). Current evidence indicates that the combination of targeted therapy and immune checkpoint inhibitors represents a rational therapeutic strategy for treatment-naïve patients with unresectable HCC. The combination of tislelizumab and lenvatinib shows promising efficacy in treating HCC. It significantly prolongs progression-free survival (PFS) and has a generally favorable tolerability profile, suggesting it as a novel therapeutic option for advanced HCC (4). However, there is limited research on relevant combination therapies. Therefore, we present a case report of an unresectable HCC patient who demonstrated a favorable clinical response to combination therapy with lenvatinib and tislelizumab, and our findings demonstrate that this conversion therapy represents a safe and effective approach for initially unresectable HCC.

## 2 Case

**Figure 1:**
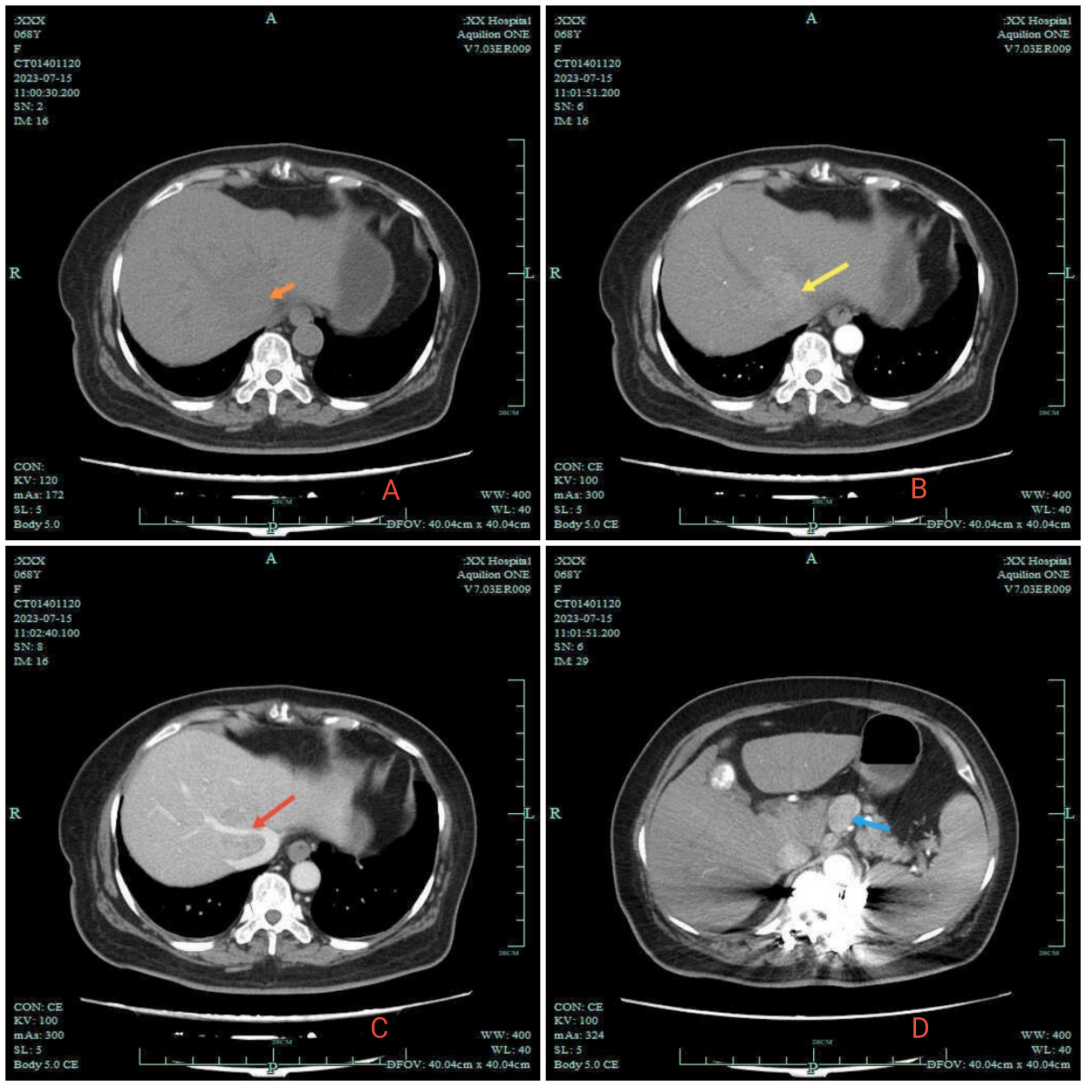
2023–7 Abdomen CT. **(A)** orange arrow in A indicates the low-density shadow of the clumps. **(B)** is the enhanced arterial phase: yellow arrow reveals significant enhancement of the tumor. **(C)** is the enhanced venous phase: red arrow shows the middle hepatic vein wrapped in the shadow of the mass. Enlarged lymph nodes are indicated by blue arrows in **(D)**.

On July 13, 2023, a 70-year-old female with fever of unknown origin was admitted to our institution. She underwent comprehensive diagnostic evaluation and management. The temperature was 38.8°C and the highest temperature after admission was 39.4°C. The patient denied associated symptoms beyond febrile episodes. Physical examination was unremarkable, with no stigmata of chronic liver disease observed. The patient had no significant family, or psychosocial history, and no known genetic disorders were documented. Notably, her medical history was negative for hepatitis B virus (HBV) infection. Laboratory tests: White blood cell count 5.8 × 10¹²/L, lymphopenia 12%, C-reactive protein 96.1 mg/L (baseline range, 0.0–5.0 mg/L). Hepatic function panel showed: total bilirubin: 7.0 umol/L, prolonged prothrombin time: 16.4 s, gamma-glutamyl transferase: 85.3 U/L (baseline range,4.0-60.0) U/L. Blood cultures yielded Staphylococcus haemolyticus. Together with other clinical and laboratory findings, this suggested hepatobiliary pathology accompanied by a systemic inflammatory response. Contrast-enhanced abdominal CT revealed several critical findings: a 2.5 × 1.9 cm irregular hyperdense lesion at the second hepatic porta; during the arterial phase, marked heterogeneous hyperenhancement was observed. Encasement of the middle hepatic vein (MHV) with luminal irregularity. And contrast-enhanced CT demonstrated significant lymphadenopathy at the porta hepatis, with multiple enlarged nodes showing a maximal short-axis diameter of 2.3 cm. Concurrently, the hepatic parenchyma exhibited multiple well-circumscribed cystic lesions, the largest measuring 1.0 cm in maximum diameter (**Figure 1**). To establish a definitive diagnosis, ultrasound-guided percutaneous liver biopsy was performed obtaining three core needle biopsies (1.6-1.8 cm in length). The liver specimen demonstrated focal fibrosis, extensive mixed inflammatory infiltrates, and coagulative necrosis. Histomorphological features suggested atypical epithelioid cell proliferation indicative of neoplastic transformation. Immunohistochemical results: Ki -67 (+, 50%), LCA (-), GS (+), Arginase -1 (-), GPC -3 (-), Hepatocyte (+), CK19 (+), CK18 (+), CK7 (+), CK (+). Combined with HIS morphology and immunohistochemical results, the diagnosis of hepatocellular carcinoma was made. Fever is a clinical manifestation of HCC. According to TNM staging, the patient’s HCC was stage II (T2NxM0), BCLC stage C, and Child-Pugh A liver function. Given suspected lymph node metastasis, systemic therapy with intravenous tislelizumab combined with oral lenvatinib was recommended following MDT consensus.

### 2.1 Treatment plan

**Figure 2:**
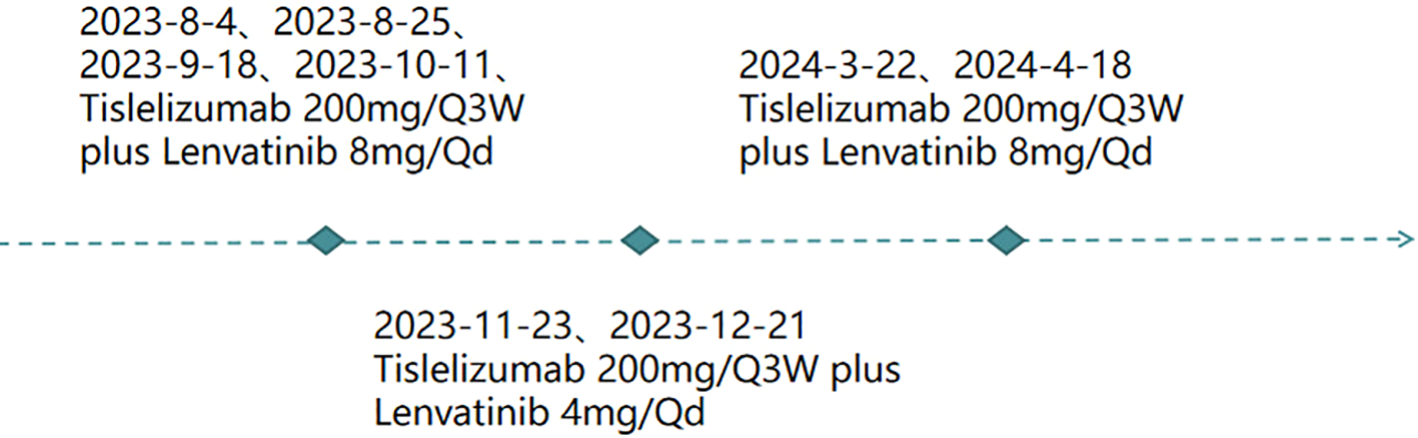
The patient’s treatment process.

①For fever: initially treated with Cefuroxime Sodium 1.5 g via IV pump Q12h. After no improvement, switched to Cefoperazone Sodium with gradual resolution of recurrent fever. ②For HCC (**Figure 2**): the patient received targeted therapy (Lenvatinib Mesylate capsules, 8 mg orally every day) plus immunotherapy (Tislelizumab, 200mg introvenously every 3 weeks) on 2023-8-4、2023-8-25、2023-9-18、2023-10-11、2023-11-23、2023-12-21、2024-3-22、2024-4-18.

### 2.2 Evolution

**Figure 3:**
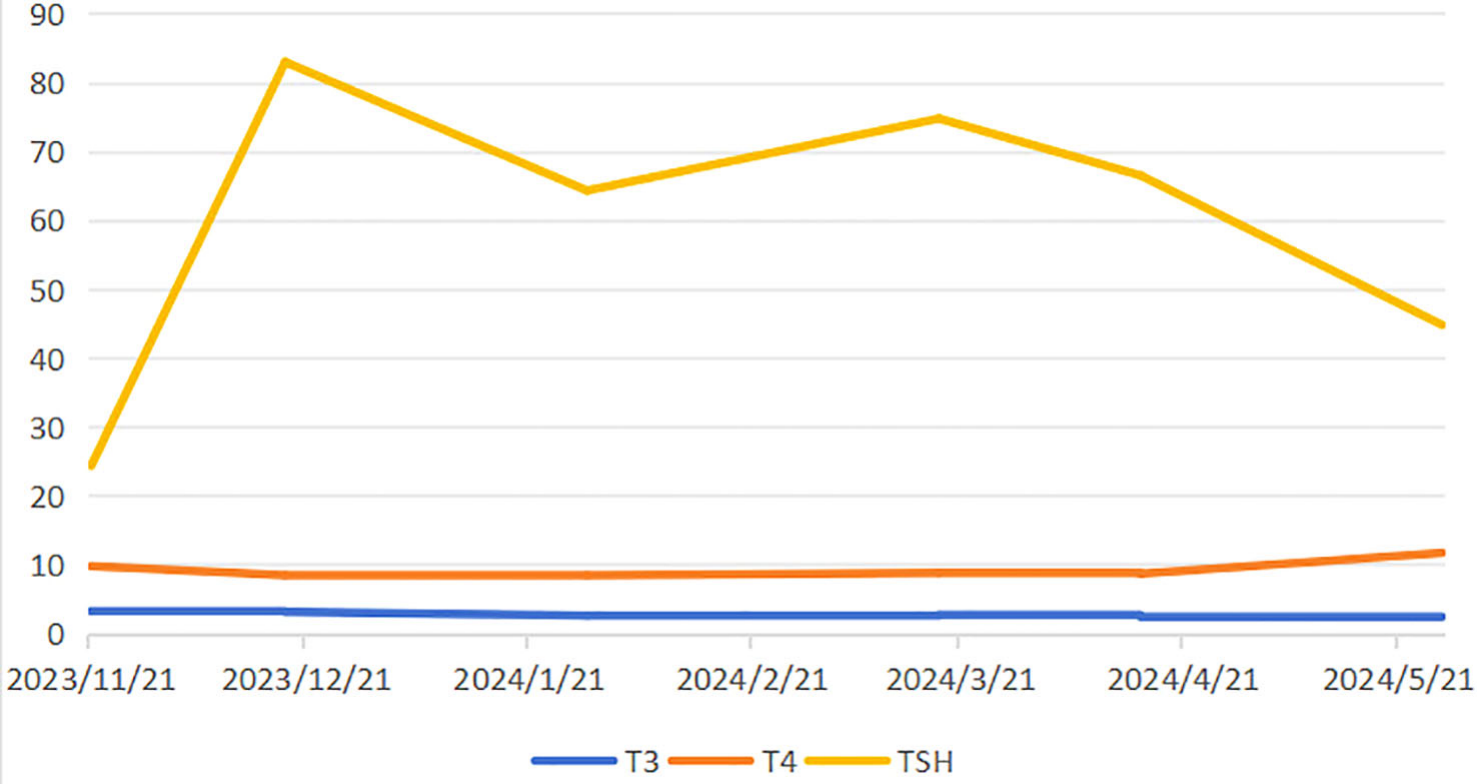
Patients with thyroid function index level changes.

**Figure 4:**
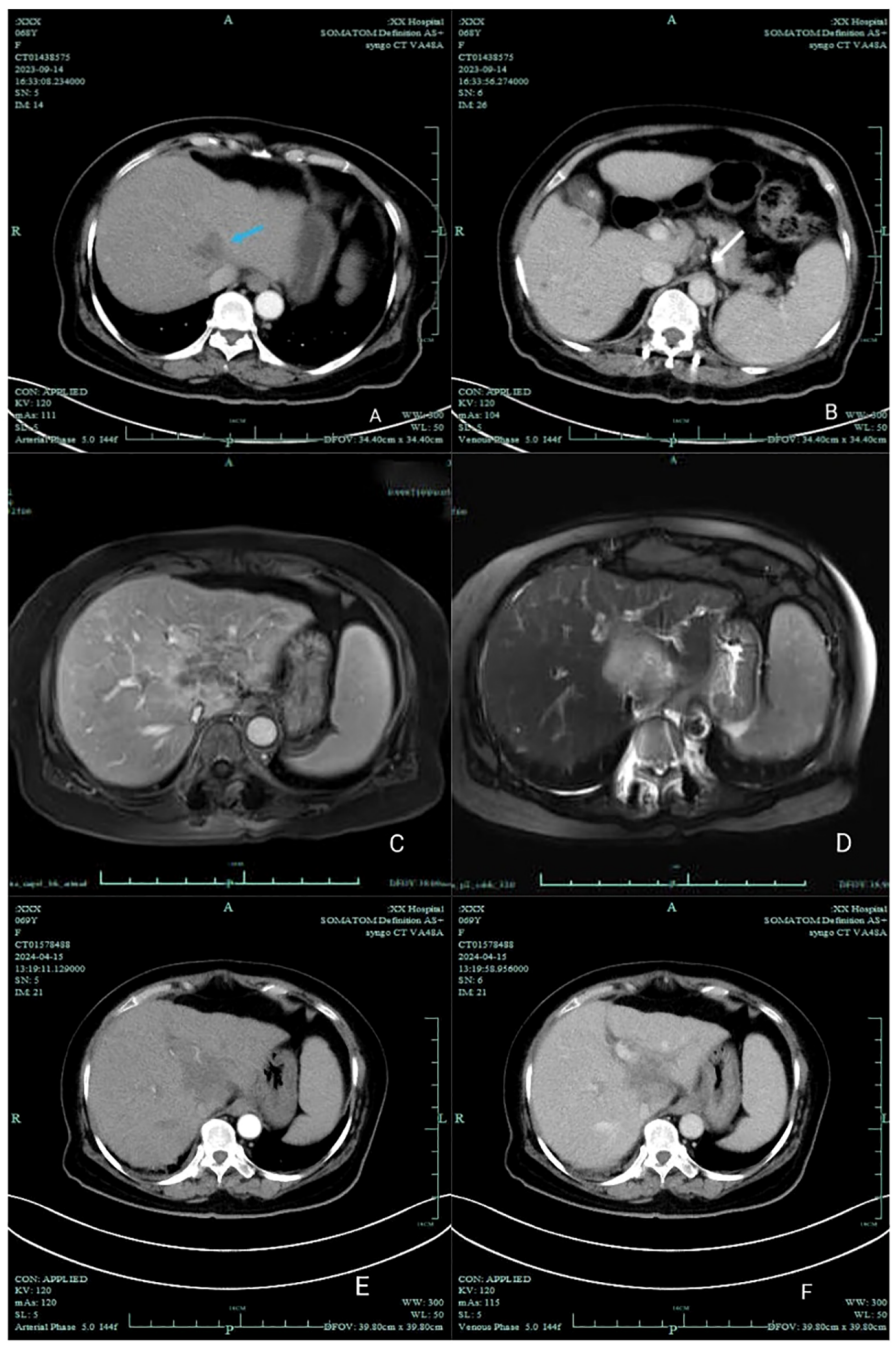
CT and MRI changes of patients. In September 2023 **(A, B)**, **(A)** showed a marked reduction in tumor vascularity. **(B)** showed multiple mildly enlarged lymph nodes around the celiac trunk. The largest lymph node measured approximately 1.0 cm in short-axis diameter, representing a 57% reduction compared to **Figure 1**. In March 2024 **(C, D)**, the tumor showed progression following targeted therapy and immunotherapy. It measured approximately 4.7 × 3.9 cm, representing an 88% increase from previous imaging. By April 2024 **(E, F)**, further tumor progression was observed, accompanied by lymph node enlargement.

After three cycles of combined targeted therapy and immunotherapy, follow-up imaging revealed a marked reduction in tumor vascularity. Additionally, there was a concomitant decrease in the size of lymph nodes around the celiac trunk compared to previous scans. Surgical intervention is now recommended. Due to the patient’s advanced age and the fact that targeted immunotherapy was well tolerated and effective, the family chose to continue this treatment and not to proceed with surgery. During systemic therapy, the patient developed therapy-related adverse events including hypertension, diarrhea, and asthenia, accompanied by laboratory abnormalities in T3, T4, and TSH levels (**Figure 3**). Bilateral thyroid lobes showed reduced tracer uptake and density on scintigraphy, indicating thyroid dysfunction. Some studies indicate that lenvatinib treatment for unresectable HCC may induce hypothyroidism (5). Based on these findings, we reduced the lenvatinib dose to 4 mg daily (half the standard dose) and started levothyroxine sodium at 25 μg orally for management of drug-induced hypothyroidism. After T3 and T4 levels normalized and fatigue resolved, the lenvatinib dose was restored to the standard regimen (8 mg once daily) with close thyroid function monitoring. Nevertheless, subsequent abdominal CT surveillance revealed rapid tumor progression with new metastatic lesions, and the patient ultimately succumbed to disease progression shortly thereafter (**Figure 4**).

## 3 Discussion

### 3.1 The mechanism of tislelizumab combined with lenvatinib

We present a case of HCC treated with tislelizumab plus lenvatinib as conversion therapy. After three cycles, tumor shrinkage was achieved; however, hypothyroidism emerged as a treatment-related adverse event. Notably, the patient and her family declined subsequent surgical intervention, precluding direct assessment of this regimen’s potential benefits on resectability or postoperative survival. Our case highlights both the antitumor efficacy and endocrine toxicity of immune-targeted combination therapy in advanced HCC.

Late-stage HCC was historically characterized by limited therapeutic advancements. However, recent progress in molecular subtyping has advanced the understanding of HCC. Additionally, the emergence of novel agents, particularly tyrosine kinase inhibitors (TKIs) and immune checkpoint inhibitors (ICIs), has revolutionized systemic therapy for advanced HCC. The median overall survival (OS) has improved from approximately 1 year to nearly 2 years, accompanied by a marked enhancement in quality of life (6). Lenvatinib, a multi-target tyrosine kinase inhibitor (TKI), exerts its antitumor effects by inhibiting the activity of vascular endothelial growth factor receptors (VEGFR1-3), fibroblast growth factor receptors (FGFR1-4), platelet-derived growth factor receptor α (PDGFRα), RET, and KIT. This blocks signaling pathways involved in tumor angiogenesis and cancer cell proliferation (7). Tislelizumab, a high-affinity humanized anti-PD-1 monoclonal antibody, exerts its antitumor effects by specifically binding to the PD-1 receptor, thereby blocking the PD-1/PD-L1 and PD-L2 signaling. This mechanism reverses tumor-mediated immune suppression and activates T cell-dependent antitumor immune responses (8). At the 2020 American Society of Clinical Oncology (ASCO) Annual Meeting, a study evaluated the combination of lenvatinib and tislelizumab in 24 HCC patients undergoing conversion therapy followed by surgical resection. The study reported an objective response rate (ORR) of 54.2% and a disease control rate (DCR) of 87.5%, highlighting the therapeutic potential of this regimen (9). A retrospective real-world study demonstrated that lenvatinib combined with programmed death-1 (PD-1) inhibitors achieved a notable median overall survival of 17.8 months in previously treated unresectable hepatocellular carcinoma (uHCC) patients (10). A phase II multicenter single-arm trial (n=64) demonstrated that tislelizumab combined with lenvatinib as first-line therapy for uHCC exhibited promising antitumor activity with favorable tolerability (11).

### 3.2 Adverse effects of treatment

Xu et al.’s study (12) included a total of 378 patients and aimed to explore the efficacy and safety of various programmed cell death protein 1 (PD-1) inhibitors combined with lenvatinib in the treatment of unresectable PLC. The most frequent treatment-related adverse events (TRAEs) included hypertension (15.1%), hyperbilirubinemia (8.5%), thrombocytopenia (6.9%), decreased appetite (6.3%), hypokalemia (6.3%), and diarrhea (5.8%), which were consistent with those reported in prior studies (4, 13, 14). The patient developed grade 1 hypertension (peak 152/77 mmHg, intermediate risk) and mild diarrhea following targeted immunotherapy; both resolved spontaneously after administration of antidiarrheal and antihypertensive medications. Notably, abdominal pain and diarrhea represent common toxicities associated with both antiangiogenic tyrosine kinase inhibitors (AA-TKIs) and immune checkpoint inhibitors (ICIs). Mechanistically, AA-TKIs may induce intestinal mucosal ischemia and hypoxia, leading to epithelial necrosis and subsequent diarrhea. Severe cases may progress to colitis requiring prompt management (15–17).

The development of fatigue in this patient was suspected to be secondary to hypothyroidism. This hypothesis was confirmed by complete symptom resolution following levothyroxine replacement therapy. To investigate the association between hypothyroidism and clinical prognosis, we conducted a systematic literature review using PubMed, CNKI, and other databases, encompassing studies from database inception to May 2024. A systematic search was performed using controlled terms and free-text keywords: (Hypothyroidism) AND (HCC). The initial search yielded 70 records, and 9 relevant articles were retained after title and abstract screening (3 Chinese and 6 English publications), indicating a limited evidence base in this field. Emerging evidence suggests that grade 2–3 hypothyroidism induced by lenvatinib therapy in advanced HCC correlates with prolonged overall survival (18). The 2024 Chinese Guidelines for Liver Cancer Management emphasize regular thyroid function monitoring in HCC patients receiving lenvatinib therapy, based on the following clinical rationale and recommendations (19). The molecular mechanism underlying TKI-induced hypothyroidism may involve suppression of thyroid-stimulating hormone receptor (TSHR) expression through the p-ERK/p-AKT–c-Myc signaling axis, leading to thyroid dysfunction. TKIs do not significantly affect TSHR transcription but likely suppress its protein expression at the post-transcriptional level. Clinical studies suggest that patients progressing to hypothyroidism demonstrate heightened TKI sensitivity compared to non-progressors, potentially correlating with improved therapeutic outcomes (20). Yang et al. (21) systematic review included 54 case reports documenting 61 adverse drug reactions (ADRs) associated with lenvatinib, with hypothyroidism reported in only one case. A retrospective study of 45 patients with unresectable or advanced HCC revealed that thyroid immune-related adverse events (irAEs) occurred in 37.8% (17 patients) of cases (22). Patients developing thyroid irAEs showed significantly prolonged survival. They also exhibited enhanced immune-mediated antitumor activity compared to those without such events. Shin et al. enrolled 208 patients with unresectable HCC treated with Ate/Bev from three Korean cancer centers. Forty-one (19.7%) of these patients experienced thyroid dysfunction, including 17.3% with hypothyroidism. They found that a portion of HCC patients treated with Ate/Bev experienced thyroid dysfunction, which was associated with favorable clinical outcomes (23). One study retrospectively analyzed data from 74 patients with HCC who received anti-PD-1 therapy. The study revealed that hypothyroidism was associated with prognosis in HCC patients treated with PD-1 inhibitors. Patients with hypothyroidism had a more favorable prognosis than those without (24). This report corroborates these findings. Therefore, continuing anticancer therapy combined with appropriate thyroid hormone replacement may contribute to prolonged survival in this setting (18). Further studies indicate that hypothyroidism may affect overall survival and alter drug therapy responses in HCC patients. For instance, hypothyroidism has been associated with longer progression-free survival (PFS) in HCC patients receiving immunotherapy. This finding aligns with the observations in this case and suggests a potentially critical role for thyroid function in treatment response (25, 26). The interaction between hypothyroidism and hepatocellular carcinoma (HCC) is complex and not fully understood. Studies show that hypothyroidism may affect the occurrence and progression of HCC by altering metabolic and endocrine functions (27). Several studies suggest that hypothyroidism may be associated with better immunotherapy outcomes. This is especially true for HCC patients treated with PD-1 inhibitors, where those with hypothyroidism show longer progression-free survival (PFS) (26).

### 3.3 Timing of surgery for therapy

The optimal timing for surgical intervention warrants further investigation. A multicenter study involving 405 patients with intermediate-advanced HCC who achieved successful conversion therapy demonstrated improved OS in the surgical resection group (n=100) compared to those receiving continued local plus systemic antitumor therapy (n=305). However, no significant benefit in event-free survival (EFS) was observed (28). A case reported by Li et al. demonstrated complete pathological remission following surgical resection in a HCC patient who underwent an 8-month course of sintilimab (PD-1 inhibitor) combined with oral lenvatinib (March to November 2023), supplemented by one session of transarterial therapy. Significant tumor reduction and normalization of tumor markers preceded surgical resection, with histopathological analysis confirming complete treatment response (29). Gyoda et al. reported that a HCC patient treated with lenvatinib demonstrated sustained disease-free survival for 51 months after treatment initiation, including 32 months following curative-intent surgery. Following 19 months of systemic therapy, the primary tumor diameter decreased to 72mm, enabling successful surgical conversion. Histopathological evaluation confirmed complete remission post-resection (30). The Chinese Expert Consensus on Conversion and Perioperative Therapy for Primary Liver Cancer (2024 Edition) recommends initiating conversion hepatectomy when tumors achieve an objective response or maintain stable disease for 3–4 months (31). When targeted-immunotherapy toxicities remain manageable, this treatment strategy can effectively stabilize intrahepatic lesions in advanced HCC and enhance survival outcomes. Post-conversion therapy assessment incorporates both radiologic response and tumor marker dynamics to guide surgical decision-making (32). Surgical intervention should be recommended for patients meeting operative criteria to improve prognosis, provided that procedural safety is rigorously ensured.

### 3.4 Limitations and future directions

In this study, combination therapy reduced tumor burden. This reduction created conditions for subsequent surgical intervention, aligning with the conversion therapy strategy reported in the literature (33). However, the limitations of combination therapy cannot be overlooked. In particular, the small sample size may affect the generalizability of the results. Although existing studies support the synergistic effect of tislelizumab and lenvatinib, most evidence derives from retrospective analyses with limited participant numbers, which may introduce bias. Furthermore, current research has not sufficiently explored the potential impact of hypothyroidism on treatment decisions, especially concerning dose adjustments in combination therapy or the use of thyroid hormone replacement therapy, highlighting a critical knowledge gap.

Despite these limitations, this study provides data on combination therapy in advanced HCC and underscores the importance of further exploring individualized treatment strategies for patients with different clinical characteristics. Additional analysis of the relationship between immunotherapy and thyroid dysfunction in HCC patients indicates that hypothyroidism may influence responses to immunotherapy. This influence likely occurs through the regulatory roles of thyroid hormones on immune cells. Specifically, existing literature suggests that thyroid hormones play a key role in modulating immune responses, especially in T cell development and function (34). Therefore, understanding the relationship between thyroid function and immunotherapy response is essential for formulating individualized treatment plans, especially when making therapeutic decisions. Future studies should focus on optimizing strategies for hypothyroid patients receiving immunotherapy to enhance treatment efficacy and reduce adverse effects.

This study has several notable limitations. First, the small sample size restricts the generalizability of the findings and may not fully represent the broader population of HCC patients with hypothyroidism. Additionally, the retrospective design relied on historical data collection, which may involve missing information and measurement errors, particularly due to inconsistent monitoring of thyroid function, potentially affecting the evaluation of treatment outcomes. The lack of a control group also precludes direct comparison between combination therapy and monotherapy, further limiting the generalizability of the findings to clinical practice. Finally, the absence of long-term data impedes a comprehensive assessment of long-term efficacy and cumulative adverse effects.

## 4 Conclusion

Our case demonstrates that lenvatinib combined with tislelizumab is effective and safe as conversion therapy for initially unresectable HCC. Because the patient was elderly and showed good tolerance and significant early response to targeted immunotherapy, the family decided to continue this regimen instead of opting for surgery. However, rapid tumor recurrence and progression occurred, and the response to further treatments was limited. This case highlights the importance of considering surgery when it becomes possible, even for elderly patients who initially respond to systemic therapy. Notably, treatment-induced hypothyroidism may serve as a potential biomarker for enhanced antitumor response. This finding underscores the critical importance of timely surgical intervention once radiologic and symptomatic improvement is achieved.

## 5 Patient perspective

The dedicated team of physicians and nurses within the hospital fosters an atmosphere of open dialogue with patients, ensures the confidentiality of their personal information, and collaborates to craft tailored treatment strategies that cater to individual needs.

## Data Availability

The original contributions presented in the study are included in the article/**Supplementary Material**. Further inquiries can be directed to the corresponding author.
